# Geriatrics Perspectives From Japan

**DOI:** 10.1093/geroni/igab046.1485

**Published:** 2021-12-17

**Authors:** Masahiro Akishita, Satoru Mochizuki

**Affiliations:** 1 The University of Tokyo, Bunkyo-ku, Tokyo, Japan; 2 Hino-Nozomi Clinic, Hino, Tokyo, Japan

## Abstract

In 2025, Japan’s baby boomers will cross the threshold of 75 years of age; a phenomenon that has been referred to as “the 2025 crisis”, resulting in a significant burden on the healthcare system. To address this issue, the Japanese government is establishing the Integrated Community Care System, to provide comprehensive medical and long-term care services in each community. In cooperation with government and affiliated organizations, the Japan Geriatrics Society (JGS) has been working to develop the Integrated Community Care System. As a result of this effort, geriatric medicine is being integrated into the health care system through incentives for practitioners. For instance, medical facilities can be reimbursed if they perform comprehensive geriatric assessments (CGA) and CGA-based management/care. Additionally, home care medicine and polypharmacy are emerging issues of interest to the government. In this symposium, I will discuss how JGS has been trying to achieve “Aging in Place” in Japan.

